# Sequence symmetry analysis graphic adjustment for prescribing trends

**DOI:** 10.1186/s12874-019-0781-1

**Published:** 2019-07-09

**Authors:** Adrian Kym Preiss, Elizabeth Ellen Roughead, Nicole Leanne Pratt

**Affiliations:** Quality Use of Medicines and Pharmacy Research Centre, School of Pharmacy and Medical Sciences, University of South Australia, Adelaide, SA Australia

**Keywords:** Sequence symmetry analysis (SSA), Waiting time distribution (WTD), Prescribing trend, Rate ratio (RR), Curve fitting

## Abstract

**Background:**

Sequence symmetry analysis (SSA) is a signal detection method that can be used to assist with adverse drug event detection. It provides both a risk estimate and a data visualisation. Published methods provide results in the form of an adjusted sequence ratio, which adjusts for underlying market trends of medicine use, however no method for adjusting the visualisation is available. We aimed to develop and evaluate another method of adjustment for prescribing trends and apply it to the SSA visualisation.

**Methods:**

The SSA method relies on incident prescriptions for pairs of medicines of interest. Smoothing curves were fitted to the frequency distributions of incident medicine use. When divided and normalised, these curves yielded a set of proportions related to differences in prescribing trends over follow-up. These were then used to adjust the unit counts for incident prescriptions in the SAA visualisation and to calculate the sequence ratio. Curve fitting was also used to identify the proportional relationship between sequences over time for SSA and is presented as a supplementary visualisation to the SSA. We compared the sensitivity and specificity of our method with that from the SSA method of Tsiropolous et al.

**Results:**

Curve-fit adjusted SSA visualisations yielded adjusted sequence ratios very close to those of Tsiropolous, with a *p*-value of 0.999 for the two sample Kolmogorov-Smirnov test. Results for sensitivity and specificity derived from adjusted sequence ratios were also practically the same. The curve-fit method graphically indicates the proportionality of the sequence and provides a useful supplement of net differences between the two sides of the SSA visualisation. Additionally, we found that incident prescriptions for patients common to both distributions are best precluded from adjustment calculations, leaving only incident prescriptions for patients unique to one or other distribution. This improved the accuracy of SSA in some atypical instances with negligible affect on accuracy elsewhere.

**Conclusions:**

Our curve-fit method is equivalent to current methods in the literature for adjusting prescribing trends in SAA, with the advantage of providing adjustment incorporated in the SAA visualisation.

**Electronic supplementary material:**

The online version of this article (10.1186/s12874-019-0781-1) contains supplementary material, which is available to authorized users.

## Background

Sequence symmetry analysis is a signal detection method used to assist with adverse drug event detection. The method is suitable for use in administrative claims data or electronic health records and has a similar performance to signal detection methods used with spontaneous adverse drug reaction reports [1–3].

SSA uses a case-only design that assesses the sequence of incident use of two medicines of interest within a specified time interval. If the use of each medicine is independent then chance alone determines the sequence order, hence the distribution of sequence orders in a population who have both medicines is effectively symmetrical. Alternatively, if a medicine is associated with an adverse event then we expect the distribution of sequence orders to be asymmetrical [4].

Specifically, if a medicine A has no adverse effect, the likelihood of starting a medicine B (as an indicator of treatment for an adverse event) after medicine A is the same as that for starting medicine B before medicine A. Where there is an adverse effect, the likelihood of starting medicine B after starting medicine A is greater than that for starting medicine B before medicine A. The method calculates the ratio of number of persons who start medicine B after medicine A, to number of persons who start medicine B before medicine A. This is called the crude sequence ratio. Because the method is sensitive to prescribing trends over time, a null-effect sequence ratio is calculated to adjust for temporal trends in medicine use. The null-effect sequence ratio is the expected sequence ratio in the absence of a causal association, given incident medicine use and events in the background population.

SSA also produces a visualisation of sequence orders, enabling the temporality of sequences to be assessed. The visualisation displays the frequency of patients by time between incident dispensing of two medicines of interest and is usually produced as a summary by weeks between sequences. It can be a valuable addition in determining the likelihood that a statistically significant result represents a signal for an adverse drug event as these are temporally associated with initiation of therapy, with the majority occurring within the first 4 months [5]. Currently, while the null-effect sequence ratio adjusts for changes in prescribing trends over time, no method is available that provides an adjusted visualisation. We aimed to develop and evaluate a method of adjustment for prescribing trends applied to the SAA visualisation.

### Waiting time distributions

**Fig. 1:**
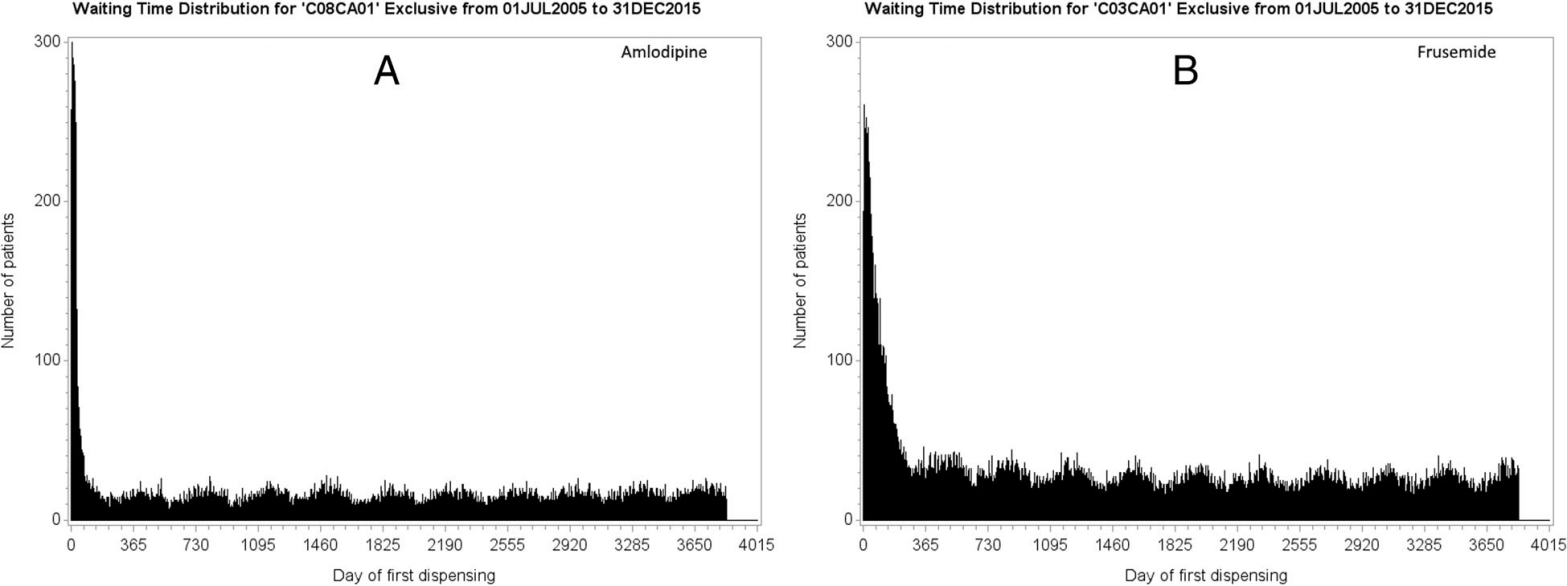
Waiting time distributions for **a**: amlodipine and **b**: frusemide over 10.5 years. The first year includes prevalent users, hence it is precluded from follow-up

An SSA visualisation is derived from two distributions of start day counts, one for each medicine, from which the patients common to both distributions are extracted. These distributions are called waiting time distributions (WTDs) [4, 6]. They are daily counts over a follow-up period for first dispensing of each medicine. Figure 1 shows WTDs for amlodipine, a medicine for hypertension or heart disease, and frusemide, a diuretic. The number of patients first dispensed each medicine was plotted for each day from data spanning 10.5 years from mid-2005 to end-2015. The higher initial counts, which, in this instance, subside within the first 12 months, include counts for repeat dispensing of medicines after unidentified dispensing sometime before mid-2005. Accordingly, the first 12 months of the WTDs is not used in the SSA as incident use in this period cannot be determined. Differences in first supply times, measured in numbers of days, between each medicine for patients common to both WTDs are called *supply lags.* These supply lags make up the SSA visualisation of sequence orders.

### Sequence symmetry analysis visualisation

**Fig. 2:**
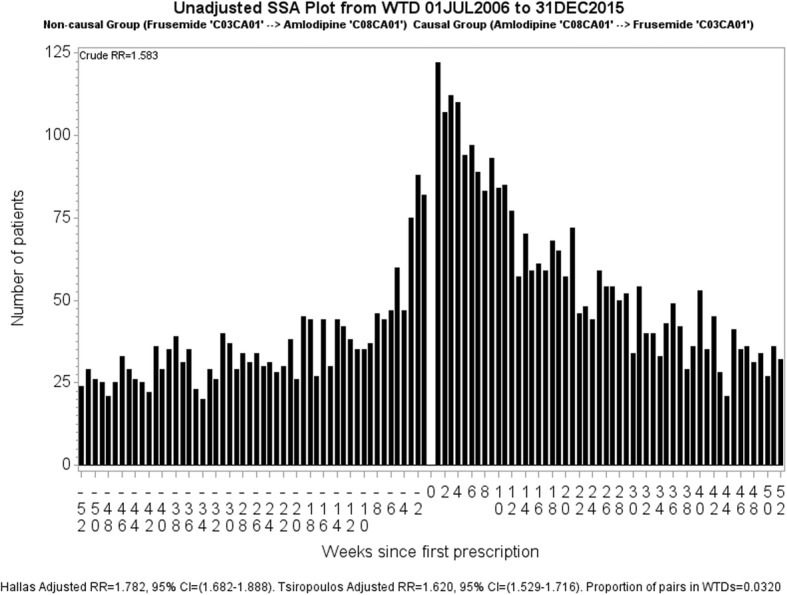
Unadjusted SSA visualisation for amlodipine and frusemide. The crude RR, 1.58, is the ratio of counts for first dispensings of frusemide occurring after amlodipine (RHS) to counts for first dispensings of frusemide occurring before amlodipine (LHS). The Tsiropolous adjusted RR is 1.62

For SSA we define a period within which we assume that the supply of a medicine may result in an adverse outcome. This period is informed by clinical relevance and indicates the period up until where we expect the medicine’s effect to have consistently diminished. Twelve months has shown to be appropriate for many medicines [1], hence our SSA visualisations are limited to 12 months. An example of an SSA visualisation is shown in Fig. 2 in which amlodipine is suspected to have an adverse effect. Each bar in the graph represents the number of patients who started frusemide in that week before or after starting amlodipine. Viewed together, the sides should show symmetry if taking one medicine does not lead to a condition that causes patients to take the other medicine. In Fig. 2 there is more incident dispensing of frusemide after incident dispensing of amlodipine than before. Because there is more than chance alone would suggest, we then conclude that some patients taking amlodipine develop a condition for which frusemide is prescribed. The obvious interpretation is that of oedema caused by vasodilatation.

The unadjusted, or crude, sequence ratio for the SSA visualisation is calculated by dividing the number of patients with incident dispensing to the right of week zero by the number of patients with incident dispensing to the left of week zero. This estimates the incidence rate ratio, which we refer to as rate ratio (RR). In Fig. 2, the crude RR is 1.58. The adjusted RR seeks to compensate for different background prescribing trends, which are changes in medicine use that have nothing to do with any adverse effect caused by a medicine but may influence the occurrence of a sequence of prescription. The two methods of adjustment adopted in the literature are Hallas [4] and Tsiropoulos [7] (see Additional file 1: Appendix 1 for more information). The Tsiropolous adjusted RR is 1.62.

The Hallas method was developed for studies where there is no limitation in the time interval between two treatments of interest. All incident observations in the study period are used. The Tsiropoulos method is an extension of the Hallas method and was developed for studies where there is an upper cap on the interval between two treatments of interest. The Tsiropoulos method applies to this study, in which it is inappropriate to make comparisons with the Hallas method.

The proportion of patients supplied both medicines, shown in Fig. 2 as proportion of pairs in WTDs, is calculated by dividing the total for patients supplied both medicines across the two WTDs by the total for all medicines (one or other medicine exclusively as well as both medicines). Over the follow-up time of 9.5 years, 3.2% of patients were supplied both medicines. This proportion is considered in the methods section.

In this paper we discuss adjustment methods for the summary statistic and introduce a new method to produce an adjusted summary statistic that also allows adjustment of the SSA visualisation. We compare the results from our method with the Tsiropoulos-style symmetry analysis, but it is implicitly adapted to the Hallas-style by not limiting the time between two treatments of interest in the SSA visualisation. In the latter instance, all incident observations in the study period are used.

## Methods

There are often differences in prescribing trends between two WTDs that need to be reconciled. If a medicine goes off patent, for example, and uptake rapidly increases, this would not be due to any causal mechanism ascribed to the other medicine and would need compensating for by adjustment to the crude RR. The adjusted RR attempts to compensate for non-causal differences between two medicines for patients common to both WTDs.

The Hallas method of adjustment was included because it provides the context from which other developments ensue. (While ensuring our software program has the flexibility to apply any of the methods where appropriate, it also allowed us to examine the methods under various test conditions.) Hallas provides a null-effect sequence ratio representing an overall adjustment, which does not adjust for counts of first supply times based on where they occur in the WTDs. Tsiropoulos’ method yields a more locally derived null-effect sequence ratio from multiple, ‘moving window’ type calculations over shorter intervals of the WTDs. This captures the effect of relatively local trend differences between two WTDs. However, neither method provides an adjustment to the shape of the SSA visualisation.

We have developed a way of calculating adjustment to the SSA visualisation by curve fitting. Curve fitting is the process of constructing a curve that fits a series of data points. It determines characteristics of the data such as rate of change anywhere on the curve, local minimum and maximum points and area under the curve (probability estimate) and can produce parameter values that most closely match the data. Most tried and proven methods of curve fitting are suitable for this purpose. (See Additional file 1: Appendix 2 for a description of the method we used.) Our default curve-fit parameter selects smoothing values half way between no smoothing and full smoothing; the former tracing the original graph and the latter tracing a straight line of some orientation.

**Fig. 3:**
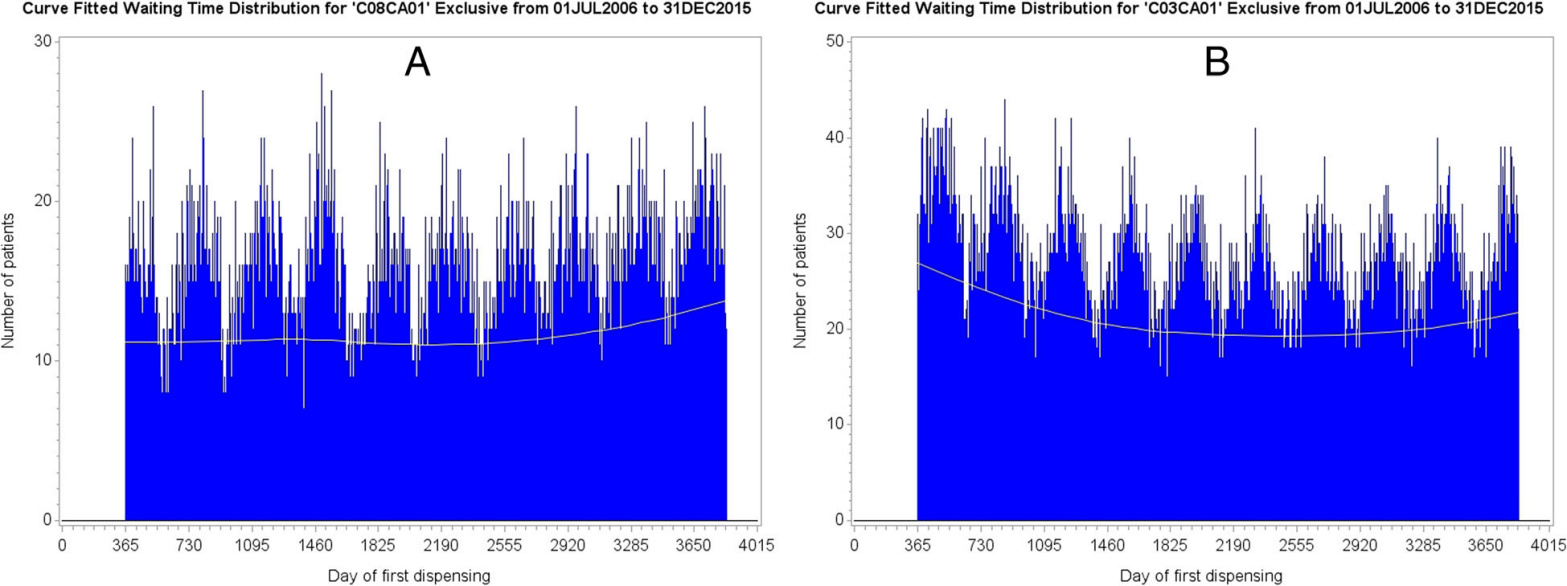
**a**: Curve fitted WTD over a 9.5-year follow-up for amlodipine (suspected cause medicine). **b**: Corresponding curve fitted WTD for frusemide (suspected effect medicine)

Upon curve fitting each WTD, as shown in Fig. 3 for amlodipine and frusemide from mid-2006 to end-2015 (first year prevalent users excluded), we then divide each fitted value of curve *B* (suspected effect medicine) by the corresponding fitted value of curve *A* (suspected cause medicine) to yield a set of ratios describing relative frequencies. The set of ratios is then normalised by dividing each value the by the average of all its values. This excludes the frequency difference between the WTDs but retains the proportional difference in shape over the WTD period. The proportions would all have a value of 1 if both WTDs had the same shape, no matter what the shape might be.

**Fig. 4:**
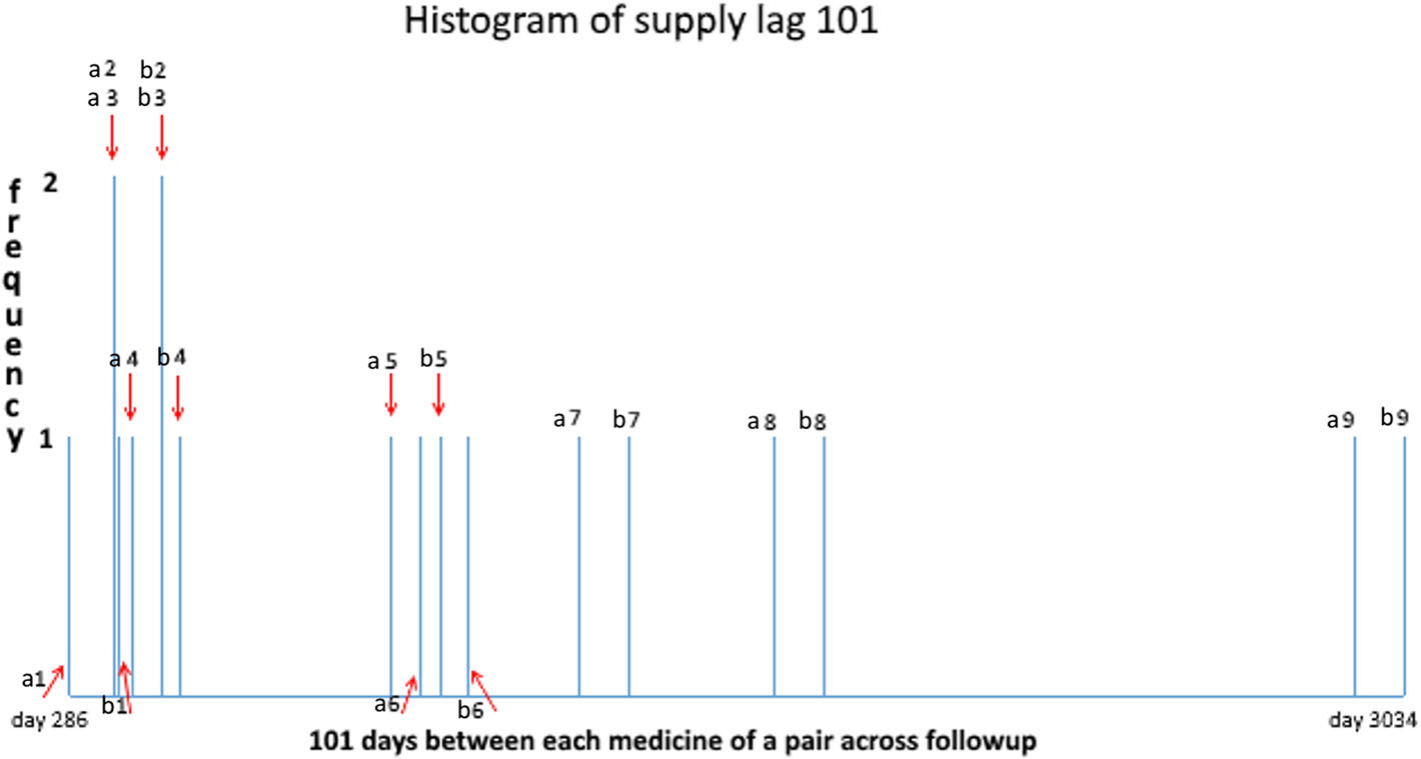
Supply lag 101 for frusemide (medicine **b**) after amlodipine (medicine **a**) across WTDs. There are nine supply lags with 101 days between each medicine of a pair. Many pairs of first supply days are unique to a patient but others can be the same for two or more patients, such as pairs 2 and 3. (Note: interleaving earlier pairs can be resolved by observing paired numbers)

As mentioned, numbers of days between supply times of each medicine for patients common to both WTDs are called supply lags*.* When accounting for underlying temporal changes in medicine use we need to consider where in the study-time supply lags occur. By way of example, Fig. 4 shows counts of all supply days over follow-up for just one of many sets of supply lags for frusemide *after* amlodipine. It shows where supply lag 101 (i.e. where frusemide was first supplied 101 days after amlodipine) occurs in the two WTDs. Counts of supply days with the same supply lags are then summed. In this instance, the count is nine. It comprises seven individual counts (frequency of 1) and two coincident counts (frequency of 2).

Referring to Table 1, there are 23 pairs of first supply days for frusemide *after* amlodipine constituting a set with supply lag 1 and 14 pairs of first supply days constituting a set with supply lag 2, and so on, out to possible supply days limited at supply lag 365. There is another such instance for frusemide *before* amlodipine for which counts of first supply days constituting sets of supply lags are also summed. These are then used to construct daily SSA data, which are further summed into weekly groupings for a weekly SSA visualisation.

**Table 1:**
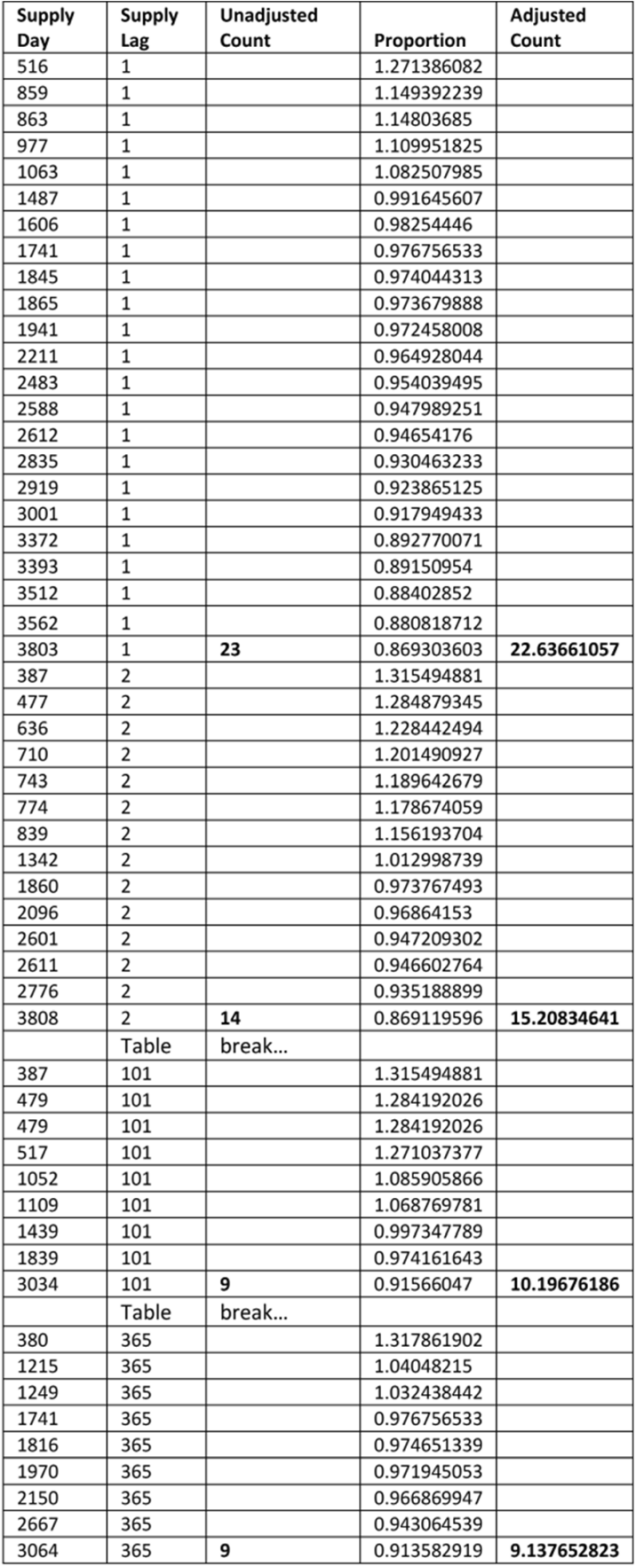
Unadjusted and adjusted counts forfrusemide-after-amlodipine supply lags 1, 2 … 101 … 365. The closer two WTDs come to the having the same shape, the closer the proportions come to having a value of one

**Fig. 5:**
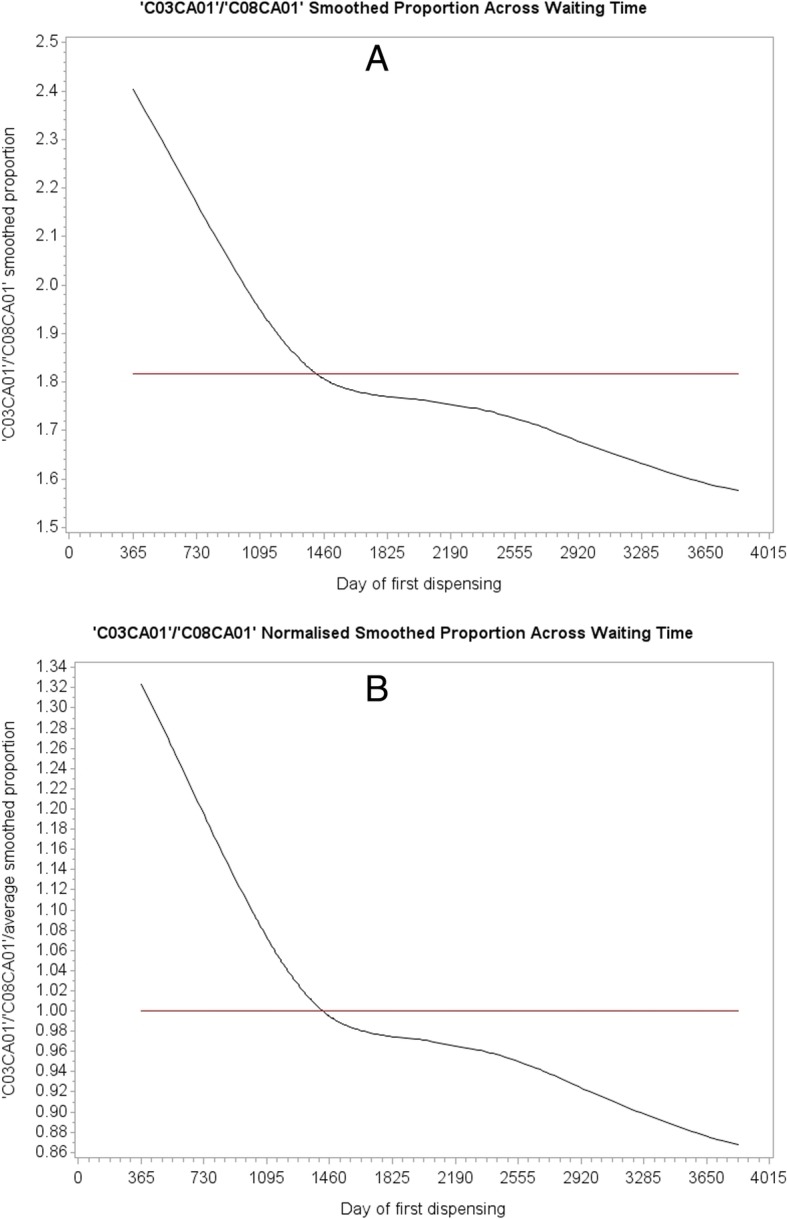
**a**: *Effect* as a proportion of *cause* due to prescribing trends. On average, 1.816 times more patients started frusemide than started amlodipine. **b**: *Effect* as a normalised proportion of *cause* due to prescribing trends. Dividing by the average (1.816) excludes the frequency difference but retains the proportional difference in shape over the WTD period

For frusemide *afte*r amlodipine there were 363 sets of supply lags, as two sets were not found in the data. Table 1 shows just four of these: 23 pairs of first supply days with a supply lag of 1 day in the WTDs, 14 with a supply lag of 2 days, 9 with a supply lag of 101 days (refer to Fig. 4), and 9 with a supply lag of 365 days. Each proportion, derived from its specific location in the two WTDs, is placed against the corresponding supply day within its supply lag set. Summing counts of each set of supply lags yields unadjusted SSA counts for the different sets and summing the proportions over these sets yields adjusted SSA counts. The same scheme applies to frusemide *before* amlodipine. This adjustment means that the unit count for each supply lag in a set is otherwise weighted by the normalised proportion according to where it occurs in the WTDs. Any day a suspected *effect* medicine of a pair occurs in its WTD, its contribution (count) is adjusted according to the relationship on that day to the suspected *cause* medicine WTD. That is, the kind of adjustment ratios on the vertical axis of Fig. 5 described by curve B, are invoked on the day in the WTDs that the* suspected effect* medicine is observed, both in the medicine B *before* A situation and the B *after* A situation.

Rate ratio adjustment compensates for different prescribing trends due to external causes. These are *changes* in prescribing frequency over time and need compensating for if each WTD is different in shape. Moreover, a medicine of interest may cause a change of prescribing frequency of another medicine, and this is additional to the underlying trend. Any such change of prescribing frequency is considered constant over follow-up; it is considered not to alter the difference between trends in the two WTDs. In practice, however, it can change over time independently in each WTD, which can be problematic if the proportion of medicine pairs is high. Hence applying any adjustment method yet discussed in the literature works best where the proportion of medicine pairs involved is typically low.

WTDs comprise exclusive components, which are supplies to persons who did not receive the other medicine of interest (i.e. not paired anywhere across the WTDs), and inclusive components, which are supplies to persons who had both medicines of interest (i.e. paired anywhere in the WTDs.) Patients can have medicine *A* exclusively, medicine *B* exclusively, or medicine *A and* medicine *B.* Where a substantial proportion of medicine pairs exist in relation to number of medicine starts in a WTD and these vary in frequency over follow-up, then this may differentially alter the original background prescribing trends between WTDs. Some of the difference in shape between two WTDs may be attributed to an effect, which would corrupt an adjustment—otherwise intended to compensate for different background prescribing trends—and tend to adjust out the effect. Consequently, only the exclusive components should be used in adjustment calculations.

**Fig. 6:**
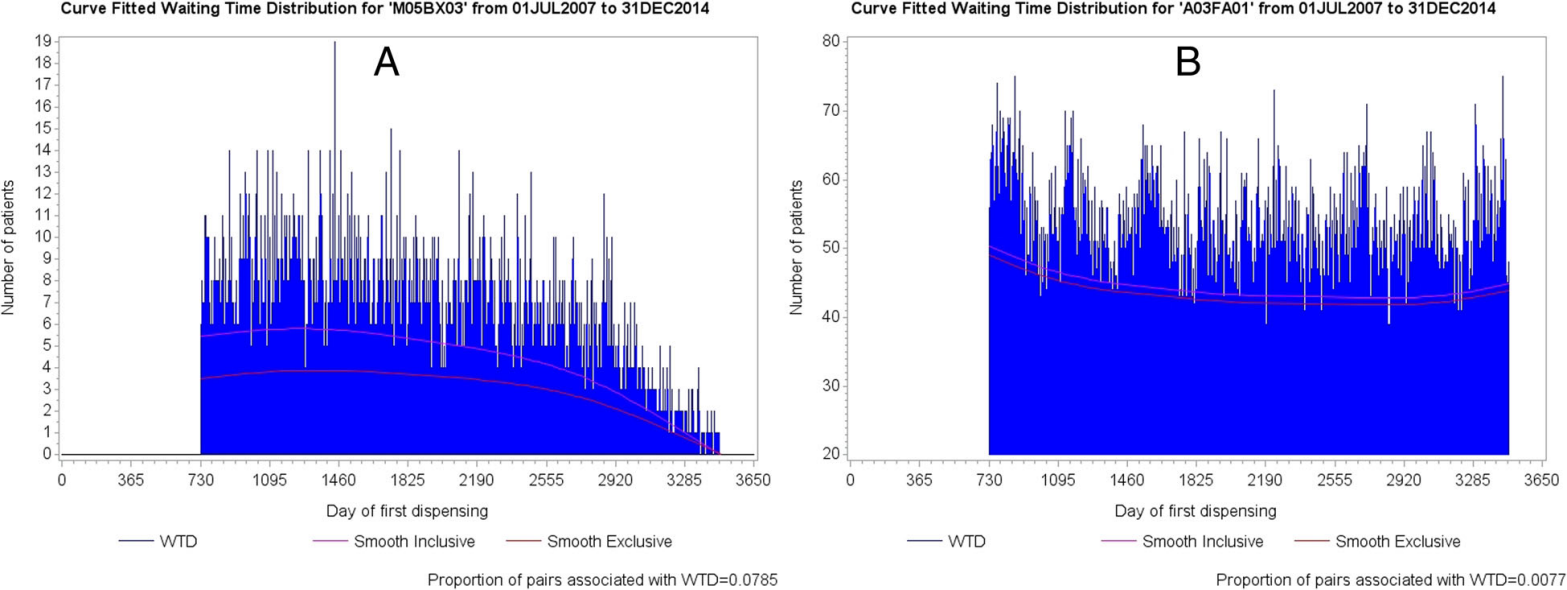
The lower curves of Figures **a** and **b** show the prescribing trend for exclusive components—true trend—and the upper curves show the prescribing trend for all components. In this example, using all components resulted in a differential change in shape between respective WTDs owing to the change in shape of the distribution at Figure **a**. For little or no change in shape between WTDs, both curves should run close to parallel within each WTD. (The two-year gap at the beginning of the WTDs indicates that fist supply times took extra time to settle down to where they could be used for reliable follow-up)

Figure 6 shows curve fitted WTDs of all components for strontium ranelate and metoclopramide, with the curve fit of exclusive components (lower curves) superposed. Although the proportion of medicine pairs across the WTDs is quite low (0.7%), the relatively low contribution to overall medicine starts from strontium ranelate means that the proportion of medicine pairs is quite high at 7.85% with respect to strontium ranelate on its own. Where there is little or no change in shape between the WTDs because a medicine of interest has little or no effect on the background prescribing trend (constant over follow-up), or because there are relatively few medicine pairs, or because there is no cause-effect association, both curves should run close to parallel within each WTD. Figure 6 shows an example where using all components of the WTDs resulted in a differential change in shape between them due to a high proportion medicine pairs for strontium ranelate.

**Fig. 7:**
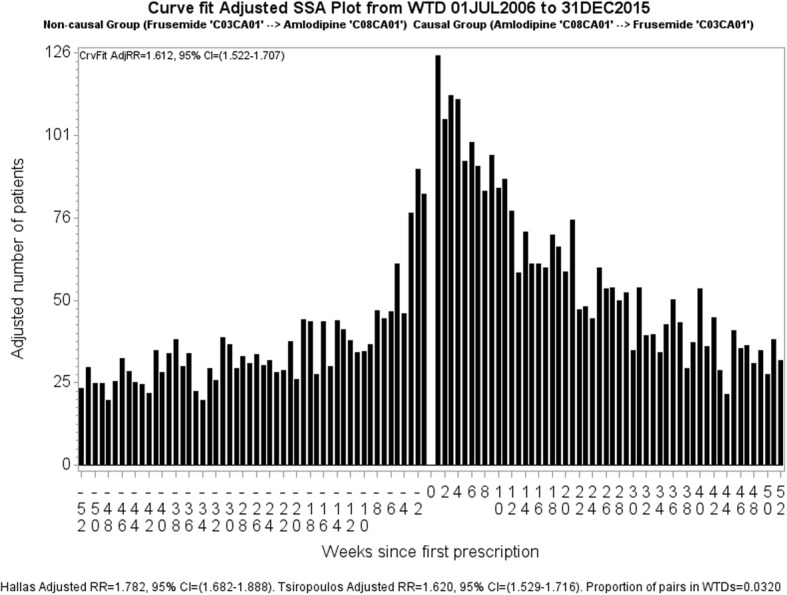
The curve-fit adjusted RR for amlodipine and frusemide is 1.61, which effectively agrees with the Tsiropoulos adjusted RR of 1.62. The proportion of patients prescribed both medicines is 3.2%

Curve fitting the distribution of *exclusive* components of each WTD eliminates this problem. It eliminates any effect signal pairs might have upon the shape of one, the other, or both WTDs, which would otherwise tend to mitigate medicine cause-effect relationships upon adjustment. Upon curve fitting exclusive components of each WTD, we then, as before, divide each fitted value of curve *B* by the corresponding fitted value of curve *A* to yield a set of ratios, which is retained across any gaps that may be left by exclusions of components. (However, adequately populated WTDs do not normally have gaps after exclusion of medicine pairs.) This is then normalised by dividing the set of ratios by its average. Figure 7 is an SSA visualisation of the curve-fit adjusted association between amlodipine and frusemide, where bar heights represent counts adjusted by the method shown in Table 1. We then calculate the curve-fit adjusted RR as before for the crude RR, by dividing the number of patients with incident dispensing to the right of week zero by the number of patients with incident dispensing to the left of week zero; only now those numbers of patients have been adjusted. Adjustment is not severe here because the shape of the two WTDs (Fig. 3, a and b) from which Figs. 2 and 7 are derived is not very different. This is confirmed by the unadjusted and adjusted RRs (1.58 vs 1.61), which are not so different.

### Supplementary visualisation

**Fig. 8:**
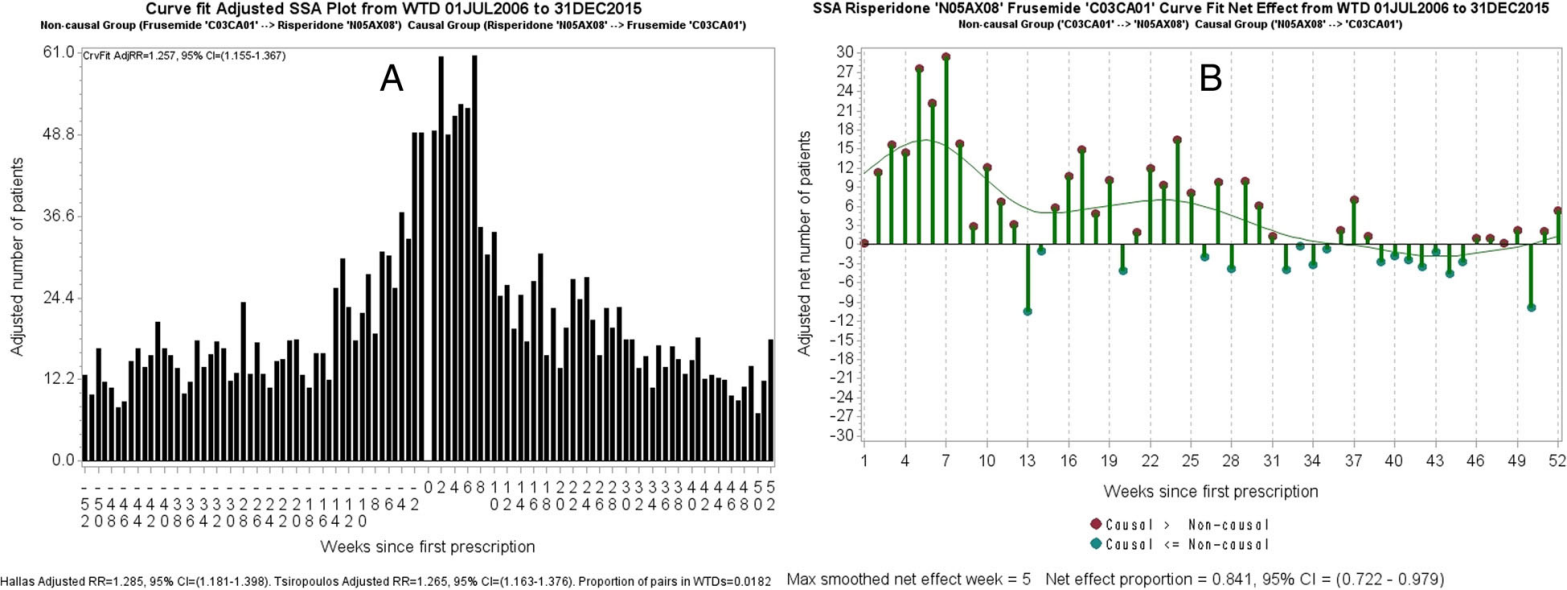
**a**: The curve-fit adjusted RR for risperidone and frusemide is 1.26, which effectively agrees with the Tsiropoulos adjusted RR of 1.27. The proportion of patients prescribed both medicines is 1.82%. **b**: The curve fitted difference where counts for after-prescribed frusemide generally exceed those for before-prescribed frusemide is greatest around week 5 and extends to week 37. After that, the counts for after-prescribed frusemide are generally less than those for before-prescribed frusemide. A sufficiently large positive effect denotes an adverse effect, a sufficiently large negative effect denotes a protective effect, and zero is the locus for no effect

Figure 8a shows a curve-fit adjusted SSA visualisation for risperidone and frusemide, and Fig. 8b shows a curve fitted visualisation of week-by-week differences between the left and right sides. We call the visualisation in Fig. 8b a ‘Net Effect’ visualisation. The *after* count for each week is subtracted from the *before* count for the corresponding week and then plotted with dots before being fitted with a curve. Red dots show the magnitudes by which weekly counts for *after*-prescribed frusemide are greater than those for *before*-prescribed frusemide and blue dots show the magnitudes by which weekly counts for *after*-prescribed frusemide are less than or equal to those for *before-*prescribed frusemide. The dots indicate spread of adjusted value differences. Net Effect visualisations show times to onset, peak and decline of an effect, and allow shape differences between each side of an SSA visualisation to be easily assessed.

The net effect proportion is calculated as the sum of positive magnitudes divided by the sums of both magnitudes. If the confidence interval for the net effect proportion straddles 0.5, then the effect is not statistically significant. Minimum and maximum possible limits for confidence intervals are implied at 0 and 1 respectively. (Of course, the net effect proportion bears a direct correspondence to the RR of the SSA visualisation.)

To validate our adjustment method, we compared it with the established method of Tsiropoulous for sensitivity and specificity to detect known adverse drug effects. The adverse drug effects assessed, along with the method, have been reported elsewhere [1]. We assessed 59 known adverse reactions (true positives) that had been identified within randomized controlled trials, and 65 negative controls (adverse effects not documented in the product information of the medicines assessed but identified as adverse effects of medicines in the other medicine classes). We used a 10% sample of persons with medicines supplied by the Australian Pharmaceutical Benefits Scheme; the national scheme that provides subsidised access to pharmaceuticals in Australia [8].

## Results

**Fig. 9:**
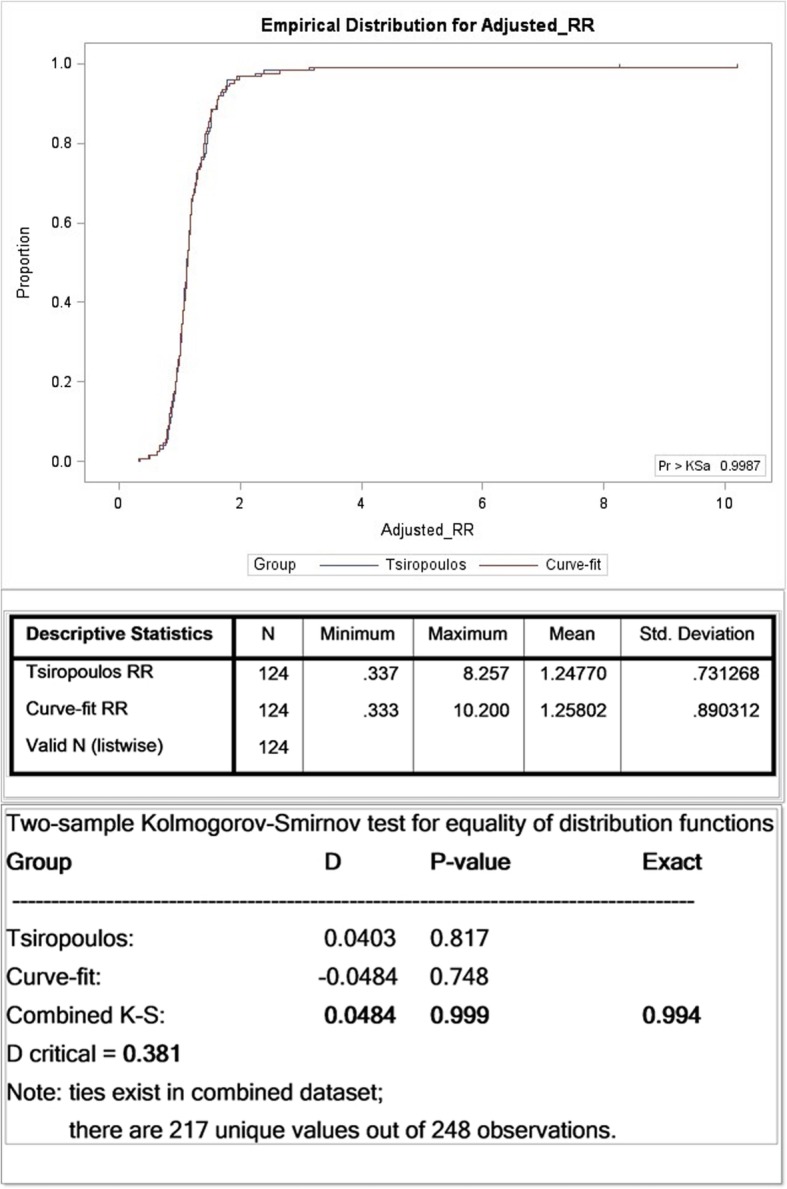
The Kolmogorov-Smirnov statistic represents distance between two samples; here adjusted RRs derived from exclusive WTD components over 9.5 years. Of 124 corresponding pairs of RRs for Tsiropoulos and Curve-fit, only a handful did not effectively coincide (four or five, depending on how an outlier is defined), and these were for grossly deficient numbers of medicine pairs, typically in single digits. The exact *p*-value for the 124 pairs of RRs was 0.994

Owing to incomplete data capture in the 10% sample, our results underestimate those normally produced by the SSA procedure [1, 2, 9]. Nonetheless the 10% sample was suitable because we were interested in relative results, not absolute results. Adjusted RRs calculated by the Tsiropoulos and Curve-fit methods are summarized in Fig. 9, which shows their respective cumulative distribution functions. The results are effectively indistinguishable with an exact *p*-value of 0.994 for the two sample Kolmogorov-Smirnov test, which tests for equality of two arbitrary distributions. (See Additional file 1: Appendix 3 for a description of the Kolmogorov-Smirnov test.) Results summarized in Table 2 for sensitivity, specificity and predictive values for the two methods over a follow-up of 9.5 years are the same. (This does not necessarily mean that every single count for a significant effect, or otherwise, is the same for each method, just that the totals are the same.)

**Table 2:**
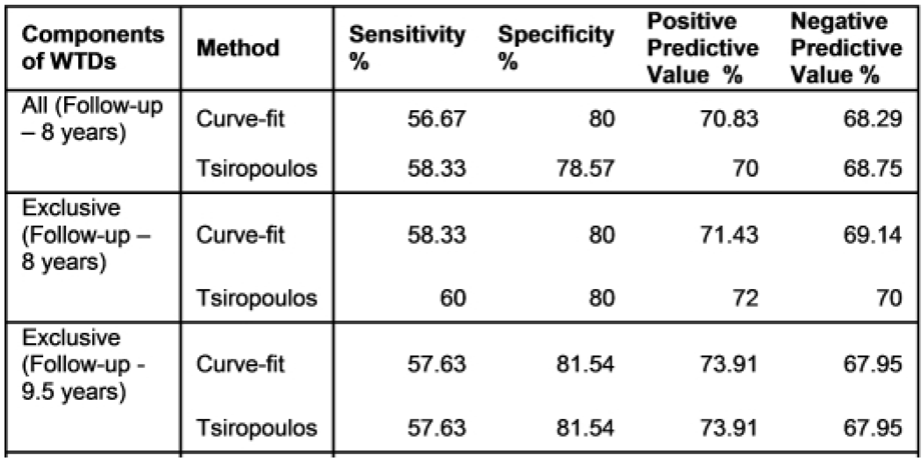
Comparison of results from 8-year WTDs show improved sensitivity, specificity and predictive values when using only exclusive components. The Curve-fit method compares closely with Tsiropoulos and yields the same result over a follow-up of 9.5 years

Having validated the Curve-fit method against Tsiropoulos, the main result is the *adjusted* SSA visualisation. The overall height of each bar for each week, representing adjusted number of patient’s first prescriptions, is derived from a composite of individual heights adjusted according to where they come from in the WTDs.

**Fig. 10:**
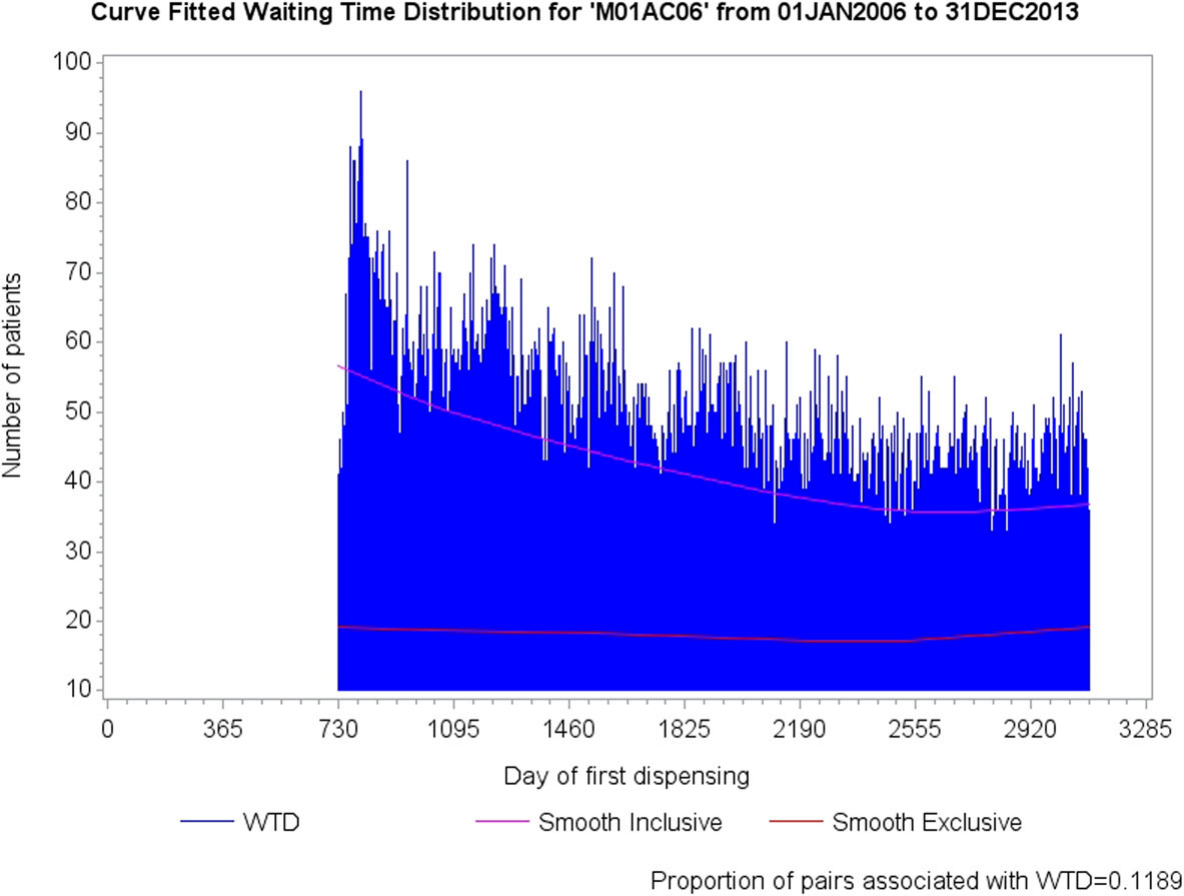
The lower curve shows the prescribing trend for exclusive components—true trend—for meloxicam and the upper curve shows the prescribing trend for all components. Again, using all components resulted in a differential change in shape between respective WTDs owing to the change in shape mainly of the meloxicam distribution

A further result derives from the use of exclusive WTD components, where we see improved sensitivity, specificity and predictive values shown in the 8-year summaries of Table 2. Table 3 shows adjusted RRs for all three adjustment methods for meloxicam and proton pump inhibitors, where the lower contribution to overall medicine starts from meloxicam means that the proportion of medicine pairs is quite high at 11.89% with respect to meloxicam on its own. The first row, derived from all components, shows confidence intervals that are not significant and the second row, derived from exclusive components, shows confidence intervals that are significant. The second row is the correct one for this medicine pair, which is known to show an adverse effect. Again, we have the situation where both curves do not run close to parallel within at least one WTD, as is evident for meloxicam in Fig. 10.

**Table 3:**
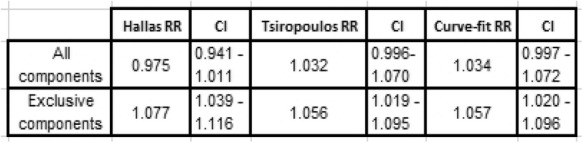
Adjusted RRs for Hallas (used advisedly here), Tsiropoulos and Curve-fit. The first row shows an incorrect result for meloxicam and proton pump inhibitors for all three methods because the confidence intervals (CI) straddle the value 1. The second row shows the correct result for all three methods

## Discussion

Needing just three variables, SSA is economical with respect to dataset size and computing resources, and is easy to deploy. It has been applied to international as well as national datasets [10]. Because it uses the experience of different persons before and after exposure, SSA cannot be considered self-controlled. However, time invariant confounders, such as age, smoking or hypertension, are effectively controlled because they do not predict prescription order given that patients become users of both drugs. That is, they do not contribute to the generation of an asymmetrical distribution of sequence orders [4, 11]. Accordingly, the SSA visualisation can usefully assess temporal relationships of effects.

SSA has proved useful as a signal detection tool to provide early alerts of drug safety issues. Results from the study by Wahab, Pratt, Wiese et al—which tested the validity of SSA to detect adverse drug reactions from an administrative claims database—show a sensitivity of 61%, specificity of 93%, positive predictive value of 77% and negative predictive value of 87% [1]. These exceed those of our study owing to the quality of the database they utilised, which afforded complete data capture in their case. Their study did not consider conditions discussed in our study that seek to further improve utility. Still, they demonstrated that SSA has the potential to identify early drug safety issues and could complement existing pharmacosurveillance methods.

We have demonstrated that curve fitting produces a similar statistic to that of the Tsiropoulos method and can be extended to provide a mechanism to adjust the visualisation of sequences. Additionally, we found that curve fitting is useful for some related procedures, such as for showing the net effect integration of each side of the SSA visualisation. Displaying net raw values also eliminates the confusion of corresponding *after* and *before* weekly comparisons sometimes necessary when viewing an SSA visualisation, by clearly showing which week is the greater or otherwise.

Not all paired observations result from a medicine effect. Remaining pairs are coincidental and become more prevalent as the RR gets closer to 1. These are no more or less important than exclusive observations. They are part of the background trend and considered to be distributed as such—hence excluding them, along with signal pairs, should not prove problematic. Curve fitting produces parameter values that closely match the data. If two curves (one for all components and one for exclusive components) displayed on the one WTD run substantially parallel, then the shape of a WTD with or without pairs remains effectively unchanged. Excluding pairs from the WTDs in the RR adjustment calculation made no effective difference in most instances in our study. However, such exclusion precludes the possibility of disproportionality that might markedly affect the shape of a WTD due to a high proportion of medicine pairs that can change over time. While an overall high number of medicine pairs with respect to all medicine starts for both WTDs can cause such a problem, an overall low number of medicine pairs can likewise do so if the frequency balance between a pair of WTDs is too one sided. For the higher frequency WTD the proportion of medicine pairs may well be low with respect its medicine starts, but for the lower frequency WTD it can be quite high. There were a few instances in which disproportionality was an issue and for these we found improved adjusted RR accuracy reflected in sensitivity, specificity and predictive values when using only exclusive components.

Finally, curve fitting, as supplied in statistical packages, is a relatively simple substitute for the mathematical complexity involved in the Tsiropoulos method. They both implement moving average procedures that capture the effect of local trend differences between two WTDs. Curve fitting also has the advantage of not having to specify a cut-off time related to the time a medicine effect may diminish. (See Additional file 1: Appendix 1, equation 3 where a cut-off time for the Tsiropoulos method must be specified.)

## Conclusions

Our curve-fit method is equivalent to the Tsiropoulos method for adjusting prescribing trends in SAA, with the advantage of providing adjustment incorporated in the SAA visualisation. An adjusted visualisation can more accurately assist in determining the likelihood that a statistically significant result represents a signal for an adverse drug event. Excluding pairs eliminates any effect signal pairs might have upon the shape of either WTD, which would otherwise tend to mitigate medicine cause-effect relationships upon adjustment. Further work is planned to test the curve-fit method on short period WTDs of about 3^1^/_2_ years to see how it compares with the Tsiropoulos method. This matters because adjustment for prescribing trends at *each end* of WTDs can be compromised due to lack of data on one or other side of an index date. The longer the WTDs, the less this is an issue. Since different statistical methods can treat boundary conditions in different ways, such a comparison is considered worthwhile.

## Additional file

Additional file 1: **Appendix 1.** Explanation of Hallas and Tsiropoulos adjustment equations for prescribing trends. **Appendix 2.** Explanation of the curve fitting method used in our study [12]. **Appendix 3.** Explanation of Kolmogorov-Smirnov test. (DOCX 26 kb)

## Data Availability

The data supporting findings of this study are available from Department of Health, Canberra, Commonwealth of Australia [8]. Restrictions apply to the availability of these data, which were used under license for this study and so are not publicly available. The licence agreement prohibits the authors from providing data to third parties. However, a suite of programs devised and used by the first author for the curve fitting method are publicly accessible at 10.25954/5ce215d095460. Copy and paste the link into a web browser. Core programs and some useful ancillary programs are supplied as is. These programs may be applied more broadly. Relevant variables, where a medicine is suspected to be associated with an adverse event, are: *1. If the adverse event is indicated by starting another medicine—*Patient identifier (or de-identifier), Primary ATC code, Medicine supply date. *2. If the adverse event is indicated by hospitalization—*Patient identifier (or de-identifier), Primary ATC code, Medicine supply date, Hospital admission date, ICD code.

## Abbreviations

RR: Rate Ratio. (Number of patients undergoing a sequence of two events, where the first is suspected to cause the second, divided by number of patients undergoing the reversed sequence of the two events.)
SSA: Sequence Symmetry Analysis. (Consecutive complementary sequences of counts for two discrete incident events; in our case within a specified time interval.)
WTDs: Waiting Time Distribution. (Two frequency distributions, each of discrete incident events in the data over the study follow-up period.)
