# Supplementary material for: Sequence symmetry analysis graphic adjustment for prescribing trends
Source: BMC Med Res Methodol. 2019 Jul 9;19:143. doi: 10.1186/s12874-019-0781-1 (PMC6617934; doi:10.1186/s12874-019-0781-1)
Supplement: Supplementary file 1 — Appendix 1. Explanation of Hallas and Tsiropoulos adjustment equations for prescribing trends. Appendix 2. Explanation of the curve fitting method used in our study [12]. Appendix 3. Explanation of Kolmogorov-Smirnov test. (DOCX 26 kb) [file 12874_2019_781_MOESM1_ESM.docx]

APPENDIX 1

For two random WTDs drawn from the same distribution the probability of drug *B* being dispensed after drug *A* depends on when drug *A* was dispensed. If drug *A* was dispensed at the latest time in the WTD then the probability of drug *B* being dispensed after drug *A* is zero; if drug *A* was dispensed at the earliest time in the WTD then the probability of drug *B* being dispensed after drug *A* is 1; if drug *A* was dispensed at the middle time in the WTD then the probability of drug *B* being dispensed after drug *A* is 0.5; and so on for any time of incident dispensing.

The numerator of equation 1 from Hallas shows the number of dispensing for drug *A* on each day of the WTDs multiplied by the number of dispensing for drug *B* on all days *after* each day on which drug *A* was dispensed. The denominator shows the number of dispensing for drug *A* on each day of the WTD multiplied by the number of dispensing for drug *B* on each day of the WTD.

Equivalent forms of Hallas:

 (1)

 (2)

where *m* and *n* index consecutive days of the WTDs and *u* is the last day of the WTDs,

*drugA_index_* is the number of patients first dispensed drug *A* on day *index,*

*drugB_index_* is the number of patients first dispensed drug *B* on day *index*.

Hence, we have the sum of products of the whole period for drug *A* and the period after day *index* for drug *B* as a proportion of the sum of products of the whole period for drug *A* and the whole period for drug *B.*

The null-effect sequence ratio is:

For two WTDs drawn from the *same* distribution, $a$ ideally equals 0.5, hence *r_n_* equals 1, therefore the crude sequence, or rate, ratio remains unchanged when multiplied by *r_n_.* However, if one WTD has a different shape with respect to the other then *r_n_* no longer equals 1. In this instance the difference in trends is said to be compensated for when the crude sequence ratio is multiplied by *r_n_.*

Tsiropoulos uses a modified version of equation 2, which more clearly shows how equation 3 was developed from equation 1. The numerator of equation 3 follows the same principle as for equation 2. However, for drug *B* he does not sum all the way to the latest time in the WTD but over some limited number of days after day *index.* The denominator follows the same principle as for the numerator. However, for drug *B* Tsiropoulos sums over the same limited number of days before day *index* and adds these to the sum of the limited number of days after day *index.* This yields a more locally derived null-effect sequence ratio. That is, *r_n_* is not derived from a proportion calculated over the whole period of the WTDs but from a proportion calculated over shorter periods of the WTDs.

Tsiropoulos:

**** (3)

Tsiropoulos’ calculation indexes from day to day within a ‘moving window’ interval traversing one WTD and a corresponding ‘moving window’ interval traversing the other WTD before and after each day of the first. While Hallas’ result derives from a global difference between two WTDs, Tsiropoulos’ result derives from localised differences.

APPENDIX 2

The interpolation method we used for smoothing was implemented in the SAS programming language by INTERPOL=SM, which specifies a smooth line is fit to data using a spline routine. It is a method for smoothing noisy data. Points on the visualisation do not necessarily fall on the line. The relative importance of plot values versus smoothness is controlled by SM=*nn*. Values for *nn* range from 0 to 99. The greater the value, the smoother the fitted curve. This produces a cubic spline that minimizes a linear combination of the sum of squares of the residuals of fit and the integral of the square of the second derivative [12].

We find a value of *nn*=50 to be suitable for all evaluations. We also find that changes away from *nn*=50, in the order of 15%, say, produce only marginally different results. Moreover, any method of producing a smoothing curve found in readily available statistical packages should be suitable.

APPENDIX 3

The *p*-value in the Kolmogorov-Smirnov test corresponds to the D statistic, which is the maximum vertical distance between the two cumulative distributions. The null hypothesis that two samples were drawn from the same distribution is rejected if the *p*-value is less than the significance level, here set at α = 0.05. Alternatively, the null hypothesis that two samples were drawn from the same distribution is rejected if D >= D critical. Exact tests, such as Monte Carlo sampling, are used in situations where asymptotic methods perform poorly. These include small data sets, large but unbalanced data sets, sparse data sets, and data sets containing ties. Because curve-fit RRs coincide closely with those of Tsiropoulos then there were ties, even at 3 significant digits. However, ties in RRs result in difficulties calculating their variance; hence the need for an exact test to confirm the asymptotic result.
